# Fast Computations for Measures of Phylogenetic Beta Diversity

**DOI:** 10.1371/journal.pone.0151167

**Published:** 2016-04-07

**Authors:** Constantinos Tsirogiannis, Brody Sandel

**Affiliations:** MADALGO and Department of Bioscience, Aarhus University, Aarhus, Denmark; Bangladesh University of Engineering and Technology, BANGLADESH

## Abstract

For many applications in ecology, it is important to examine the phylogenetic relations between two communities of species. More formally, let T be a phylogenetic tree and let *A* and *B* be two samples of its tips, representing the examined communities. We want to compute a value that expresses the phylogenetic diversity between *A* and *B* in T. There exist several measures that can do this; these are the so-called phylogenetic beta diversity (*β*-diversity) measures. Two popular measures of this kind are the Community Distance (CD) and the Common Branch Length (CBL). In most applications, it is not sufficient to compute the value of a beta diversity measure for two communities *A* and *B*; we also want to know if this value is relatively large or small compared to all possible pairs of communities in T that have the same size. To decide this, the ideal approach is to compute a standardised index that involves the mean and the standard deviation of this measure among all pairs of species samples that have the same number of elements as *A* and *B*. However, no method exists for computing exactly and efficiently this index for CD and CBL. We present analytical expressions for computing the expectation and the standard deviation of CD and CBL. Based on these expressions, we describe efficient algorithms for computing the standardised indices of the two measures. Using standard algorithmic analysis, we provide guarantees on the theoretical efficiency of our algorithms. We implemented our algorithms and measured their efficiency in practice. Our implementations compute the standardised indices of CD and CBL in less than twenty seconds for a hundred pairs of samples on trees with 7 ⋅ 10^4^ tips. Our implementations are available through the R package PhyloMeasures.

## Introduction

Ecologists often distinguish three kinds of diversity. Alpha diversity describes the diversity of one sample (such as the number of plant species in a vegetation plot), beta diversity describes the dissimilarity between a pair of samples, and gamma diversity describes the diversity of a large set of samples [1, 2]. These concepts can be applied to a number of diversity measures, including species richness, functional diversity and phylogenetic diversity [3, 4]. Phylogenetic beta diversity describes the phylogenetic distance among pairs of communities [5]. It is an increasingly widely-used concept in ecology, with many interesting recent applications, for example in biogeographical regionalization [6] and understanding broad-scale species distributions [7, 8]. Accordingly, there has also been a recent proliferation of methods to describe beta diversity [9].

Recent work on phylogenetic diversity has made great progress in developing efficient approaches to calculating alpha diversity measures (e.g. the works by Steel, O’Dwyer et al., Tsirogiannis et al., Nipperess and Matsen, Chao et al. [10–15]). On the other hand, so far there are no known algorithms that can efficiently compute phylogenetic beta diversity on large numbers of samples given large phylogenetic trees. This limits their practical applications, and the problem will grow as ecologists analyze larger and larger trees and numbers of samples. To fulfill their potential in a world of growing datasets, it is important that phylogenetic beta diversity metrics can be computed efficiently. Here, we focus on two widely-used metrics related to beta diversity. The first, Community Distance (CD), is the beta diversity analog of the Mean Pairwise Distance (MPD) alpha diversity measure. The second, Common Branch Length (CBL) is not strictly speaking a beta diversity measure (because it describes similarity, rather than difference, between two samples), but it is intimately related to two frequently-used measures, PhyloSor and UniFrac, of which UniFrac is a measure of dissimilarity and PhyloSor can be easily converted to one using 1-PhyloSor [9]. Our primary goal is to develop methods to compute these measures efficiently even on very large trees.

A notable lack in the literature on phylogenetic beta diversity measures is a correction for the species richness of the samples. With respect to phylogenetic alpha diversity measures such as the Mean Pairwise Distance (MPD), the Mean Nearest Taxon Distance (MNTD), and the Phylogenetic Diversity (PD), it is well-known that these measures depend on species richness, and so it is common practice to compute an index that standardizes these values against the species richness of the sample using one of a number of possible null models [16, 17]. This index combines the original value of the measure for a species sample *K* on a given tree T, with the mean and the variance of this measure’s value among all species samples in T that have the same number of elements as *K*. However, despite the dependencies of phylogenetic beta diversity measures on species richness, such corrections are not usually applied, perhaps because of the difficulty in standardizing against two species richness values simultaneously. And when such corrections are attempted, they involve slow and imprecise randomization tests [8, 18–20]. Hence, a second goal is to allow such standardization for phylogenetic beta diversity measures by developing algorithms to efficiently and precisely compute the expectation and variance of a beta diversity measure given a tree and pair of species richness values. This expectation and variance depend on a null model which describes the probability of drawing any particular pair of species sets with the specified species richness values. Here, we consider the case that all such pairs are equally probable. It is also possible to define a variety of unequal-probability cases, where the probability can depend on species abundances [21].

Care must be exercised in the interpretation of phylogenetic beta diversity measures, particularly CD and its standardised version [22]. In particular, CD is not a suitable dissimilarity measure, as the CD for a community A and itself is generally not zero, and may be greater than the CD between A and a different community B. As long as this limitation is understood, the measure can still be a useful description of phylogenetic beta diversity in some contexts (e.g. as indicated in the works of Swenson et al. and Pellisier et al. [4, 20]). And, as with measures of standardized alpha diversity, it is crucial to carefully consider the appropriate species pool upon which to base the standardization [5]. When considered carefully, however, the dependence on pool size may actually prove to be a useful feature, allowing more nuanced ecological inference [21, 23], as one allows pool size to vary.

In the present work, we provide results that lead to efficient computations of the the beta diversity measures CD and CBL, and their standardized indices. First, given a phylogenetic tree T, we show how to derive analytical expressions for computing exactly the mean and the variance of the CD and the CBL among pairs of species samples in T that have given sizes. Based on these expressions, for each of these two measures we describe efficient algorithms for computing the measure’s standardized index for a given pair of samples (and also the measure’s original value for this pair). These algorithms are developed based on standard techniques in Algorithms Design, and we provide theoretical guarantees for their performance. We implemented all of the algorithms the we describe, and we conducted experiments that exhibit their efficiency in practice. We measured the performance of our algorithms on trees of several sizes, extracted from a phylogeny that has 71,181 tips and 83,751 nodes in total. As we show later in detail, our implementations ran very fast even for the largest trees that we considered; for each of the two measures, our programs managed to compute in less than a minute the standardized indices for one hundred pairs of species samples on the complete tree with the 71,181 tips. We have made the programs that we developed publicly available through the open source R package PhyloMeasures [24]. To exhibit the strength of fast beta diversity computations, we also present an application example; we compute heat maps that illustrate the beta diversity values between a focal point assemblage and assemblages distributed on a world grid. Given our efficient algorithms, it is now possible to produce several such maps in high resolution, something that was infeasible with previously existing software.

## Analysis

### Terminology and Notation

Let T be a phylogenetic tree. We use *V* to indicate the set of nodes (representing the species/taxa) in T, and we use *E* to denote the set of edges (links between nodes) in this tree. For an edge *e* ∈ *E*, we denote the (always positive) weight of this edge by *w*_*e*_. Depending on the context, the edge weights in T may represent time intervals, molecular distance, or some other notion of difference between taxa. The analysis in this paper does not depend on this notion of difference. We denote the set of leaf nodes in T (taxa that do not have any child species) by *S*. We refer to these nodes as the *tips* of T. We indicate the number of the tips in T by s(T) or simply *s*, and we indicate the number of all the nodes in T by *n*. We consider that T is a rooted tree.

For any edge *e* be in T, we use Ch(*e*) to denote the edges that are adjacent to the child node of *e*. We call these edges the *children* of *e*. Let *u* be a node in T, and let *e* be the edge that connects *u* with its parent node. We use interchangeably T(u) and T(e) to denote the subtree of T whose root is *u*. We use *S*(*u*) and *S*(*e*) to indicate the set of tips that appear in T(u). We denote the number of these tips by *s*(*u*) and *s*(*e*).

Let *u*, *v* be two nodes in T. We call a *simple path* between these nodes the cycle-free sequence of edges that we have to traverse in order to reach *u* from *v*. We call the *cost* of this path the sum of the weights of all the edges in the path. We denote this cost by *cost*(*u*, *v*). Since T is a tree, there exists a unique simple path between any pair of nodes in T. We call the *height* of T the maximum number of edges that appear on a simple path between the root of T and any leaf. We represent the height of T by h(T). Let *R* ⊆ *S* be any sample (subset) of the tips in T. We denote the number of tips in this sample by |*R*|. We indicate the set of all paths that connect two elements in *R* by Paths(*R*), that is:

$$\begin{array}{c} \text{Paths}(R)=\{p(u,v):u,v\in R\} \end{array}$$

We denote the set whose elements are all subsets of *S* that have cardinality exactly *r* by Sub(*S*, *r*). For an edge *e* ∈ *E* and a subset *R* of the tips of T, we denote the elements of *S*(*e*) that are also elements of *R* by *S*_*R*_(*e*), that is *S*_*R*_(*e*) = *S*(*e*)∩*R*. We indicate the the number of these tips as *s*_*R*_(*e*).

For a given phylogenetic tree T we call the *total path cost of* T the sum of the costs of all distinct simple paths that connect tips of T. We denote this quantity by TC(T). Thus, the toal path cost of a tree T is equal to:

$$\begin{array}{c} TC\left(T\right)=\sum_{\{u,v\}\in S}cost\left(u,v\right). \end{array}$$

Let *e* be an edge of T. We call the *total path cost of* *e* the sum of the costs of all simple paths in Paths(*S*) that contain *e*. We denote this quantity by *TC*(*e*), thus:

$$\begin{array}{c} TC\left(e\right)=\sum_{\begin{array}{c} \{u,v\}\in S\\ e\in p(u,v) \end{array}}cost\left(u,v\right). \end{array}$$

For a node *u* that is a tip of T, we call the *total path cost of* *u* the sum of the costs of all simple paths between *u* and any other tip of T. We indicate this quantity by *TC*(*u*). Note that *TC*(*u*) = *TC*(*e*), where *e* is the edge adjacent to *u*.

### The Community Distance

Let T be a phylogenetic tree, and let *A*, *B* ⊆ *S* be two samples of its tips with |*A*| = *a*, and |*B*| = *b*. The *Community Distance* (CD) between *A* and *B* is equal to the sum of the costs of all paths that connect a tip in *A* with a tip in *B*, divided by the total number of these paths. Therefore, the community distance between *A* and *B* is equal to:

$$\begin{array}{c} \text{CD}\left(T,A,B\right)=\frac{1}{ab}\sum_{u\in A}\sum_{v\in B}cost\left(u,v\right). \end{array}$$

Samples *A* and *B* may not necessarily be of equal size. **The CD is analogous to the** ***Mean Pairwise Distance*** **measure for computing the average distance between two samples of species**.

#### The *β* Net Relatedness Index

Given a phylogenetic tree T and two samples of its tips *A*, *B* such that |*A*| = *a* and |*B*| = *b*, the standardized index of the CD for these samples is equal to:

$$\begin{array}{c} {\text{NRI}}_{\beta }\left(T,A,B\right)=\frac{\text{CD}\left(T,A,B\right)-E_{\text{CD}}\left(T,a,b\right)}{sd_{\text{CD}}\left(T,a,b\right)}, \end{array}$$ (1)

where $E_{\text{CD}}\left(T,a,b\right)$ and $sd_{\text{CD}}\left(T,a,b\right)$ are respectively the expectation and the standard deviation of the CD for all pairs of tip samples such that one sample contains *a* tips and the other sample contains *b* tips. The following theorem provides an analytical expression for the expectation of the CD.

**Theorem 1**. *Let* T *be a phylogenetic tree that has s tips, and let a, b be positive integers with a, b ≤ s. The expected value of the CD among all pairs of tip samples in* T, *such that one sample consists of a tips, and the other consists of b tips, is equal to*:

$$\begin{array}{c} E_{\text{CD}}\left(T,a,b\right)=\frac{2}{s^{2}}\cdot TC\left(T\right) \end{array}$$

*Proof*. The expectation of the CD is equal to:

$$\begin{array}{ccc} & & E_{\text{CD}}\left(T,a,b\right)=\\ & & \;E_{\begin{array}{c} A\in \text{Sub}(S,a)\\ B\in \text{Sub}(S,b) \end{array}}\left[\frac{1}{ab}\sum_{\{u,v\}\in S}cost\left(u,v\right)\cdot \left(AP_{A}\left(u\right)\cdot AP_{B}\left(v\right)+AP_{A}\left(v\right)\cdot AP_{B}\left(u\right)\right)\right]=\\ & & \;\frac{1}{ab}\sum_{\{u,v\}\in S}cost\left(u,v\right)\cdot E_{\begin{array}{c} A\in \text{Sub}(S,a)\\ B\in \text{Sub}(S,b) \end{array}}\left[AP_{A}\left(u\right)\cdot AP_{B}\left(v\right)+AP_{A}\left(v\right)\cdot AP_{B}\left(u\right)\right], \end{array}$$

where *AP*_*A*_(*u*) is a random variable such that *AP*_*A*_(*u*) = 1 if *u* ∈ *A*, otherwise its value is zero. It holds that:

$$\begin{array}{ccc} & & E_{\begin{array}{c} A\in \text{Sub}(S,a)\\ B\in \text{Sub}(S,b) \end{array}}\left[AP_{A}\left(u\right)\cdot AP_{B}\left(v\right)+AP_{A}\left(v\right)\cdot AP_{B}\left(u\right)\right]=\\ & & \;2\cdot E_{\begin{array}{c} A\in \text{Sub}(S,a)\\ B\in \text{Sub}(S,b) \end{array}}\left[AP_{A}\left(u\right)\cdot AP_{B}\left(v\right)\right]=\frac{2ab}{s^{2}}, \end{array}$$

which yields the analytical expression for the expectation of the CD measure.

Next we present an analytical expression for calculating the standard deviation of the CD measure.

**Theorem 2**. *Let* T *be a phylogenetic tree that has s tips, and let a, b be positive integers with a, b ≤ s. The standard deviation of the CD among all pairs of tip samples in* T, *such that one sample consists of a tips, and the other consists of b tips, is equal to*:

$$\begin{array}{c} \sqrt{k_{1}\cdot TC^{2}\left(T\right)+\left(k_{2}-k_{1}\right)\sum_{u\in S}TC^{2}\left(u\right)+\left(k_{1}-2k_{2}+k_{3}\right)\sum_{e\in E}w_{e}\cdot TC\left(e\right)-E_{\text{CD}}^{2}\left(T,a,b\right)}, \end{array}$$

*where*: $k_{1}=\frac{4(a-1)(b-1)}{abs^{2}{\left(s-1\right)}^{2}}$, $k_{2}=\frac{2(a-1)(b-1)}{abs^{2}{\left(s-1\right)}^{2}}+\frac{b-1}{abs^{2}\left(s-1\right)}+\frac{a-1}{abs^{2}\left(s-1\right)}$, $k_{3}=\frac{2(a-1)(b-1)}{abs^{2}{\left(s-1\right)}^{2}}+\frac{2}{abs^{2}}$.

*Proof*. The standard deviation of the CD is equal to:

$$\begin{array}{c} \sqrt{E_{\begin{array}{c} A\in \text{Sub}(S,a)\\ B\in \text{Sub}(S,b) \end{array}}\left[{\text{CD}}^{2}\left(T,A,B\right)\right]-E_{\text{CD}}^{2}\left(T,a,b\right)}. \end{array}$$

We already provided an analytical expression for the expectation of the CD; hence, we now focus on deriving an expression for the expectation of the squared value of this measure. We get that:

$$\begin{array}{ccc} & & E_{\begin{array}{c} A\in \text{Sub}(S,a)\\ B\in \text{Sub}(S,b) \end{array}}\left[{\text{CD}}^{2}\left(T,A,B\right)\right]=\\ & & \;E_{\begin{array}{c} A\in \text{Sub}(S,a)\\ B\in \text{Sub}(S,b) \end{array}}\left[\frac{1}{a^{2}b^{2}}\sum_{\{u,v\}\in S}\sum_{\{x,z\}\in S}cost\left(u,v\right)\cdot cost\left(x,z\right)\cdot P\left(u,v,A,B\right)\cdot P\left(x,z,A,B\right)\right]=\\ & & \;\frac{1}{a^{2}b^{2}}\sum_{\{u,v\}\in S}\sum_{\{x,z\}\in S}cost\left(u,v\right)\cdot cost\left(x,z\right)\cdot E_{\begin{array}{c} A\in \text{Sub}(S,a)\\ B\in \text{Sub}(S,b) \end{array}}\left[P\left(u,v,A,B\right)\cdot P\left(x,z,A,B\right)\right], \end{array}$$ (2)

where $P\left(u,v,A,B\right)=AP_{A}\left(u\right)\cdot AP_{B}\left(v\right)+AP_{A}\left(v\right)\cdot AP_{B}\left(u\right)$.

From the last expression we get:

$$\begin{array}{ccc} & & E_{\begin{array}{c} A\in \text{Sub}(S,a)\\ B\in \text{Sub}(S,b) \end{array}}\left[P\left(u,v,A,B\right)\cdot P\left(x,z,A,B\right)\right]=\\ & & \;\{\begin{array}{cc} \frac{4a(a-1)b(b-1)}{s^{2}{\left(s-1\right)}^{2}} & \text{if}\;\{u,v\}\cap \{x,z\}=\emptyset .\\ \frac{2a(a-1)b(b-1)}{s^{2}{\left(s-1\right)}^{2}}+\frac{a(a-1)b}{s^{2}\left(s-1\right)}+\frac{ab(b-1)}{s^{2}\left(s-1\right)} & \text{if}\;|\{u,v\}\cap \{x,z\}|=1.\\ \frac{2a(a-1)b(b-1)}{s^{2}{\left(s-1\right)}^{2}}+\frac{2ab}{s^{2}} & \text{if}\;\{u,v\}=\{x,z\}. \end{array} \end{array}$$

Thus, we can rewrite Eq (2) as follows:

$$\begin{array}{ccc} & & k_{1}\sum_{\{u,v\}\in S}\sum_{\begin{array}{c} \{x,z\}\in S\\ \{u,v\}\cap \{x,z\}=\emptyset \end{array}}cost\left(u,v\right)\cdot cost\left(x,z\right)\\ & & \;+k_{2}\sum_{\{u,v\}\in S}\sum_{\begin{array}{c} \{x,z\}\in S\\ |\{u,v\}\cap \{x,z\}|=1 \end{array}}cost\left(u,v\right)\cdot cost\left(x,z\right)+k_{3}\sum_{\{u,v\}\in S}cost^{2}\left(u,v\right), \end{array}$$ (3)

where $k_{1}=\frac{4a(a-1)b(b-1)}{s^{2}{\left(s-1\right)}^{2}}$, $k_{2}=\frac{2a(a-1)b(b-1)}{s^{2}{\left(s-1\right)}^{2}}+\frac{a(a-1)b}{s^{2}\left(s-1\right)}+\frac{ab(b-1)}{s^{2}\left(s-1\right)}$, and $k_{3}=\frac{2a(a-1)b(b-1)}{s^{2}{\left(s-1\right)}^{2}}+\frac{2ab}{s^{2}}$.

Next, we simplify the expression that appears in Eq (3). The last of the three sums in this expression can be rewritten as:

$$\begin{array}{c} \sum_{\{u,v\}\in S}cost^{2}\left(u,v\right)=\sum_{\{u,v\}\in S}\sum_{e,l\in p(u,v)}w_{e}\cdot w_{l}=\sum_{\{u,v\}\in S}\sum_{e,l\in p(u,v)}w_{e}\cdot w_{l}= \end{array}$$ (4)

$$\begin{array}{c} \sum_{e\in E}w_{e}\sum_{\begin{array}{c} p(a,b)\in \text{Paths}(S)\\ e\in p(a,b) \end{array}}\sum_{l\in p(a,b)}w_{l}=\sum_{e\in E}w_{e}\cdot TC\left(e\right) \end{array}$$ (5)

The second double sum in Eq (3) can be simplified as follows:

$$\begin{array}{ccc} & & \sum_{\{u,v\}\in S}\sum_{\begin{array}{c} \{x,z\}\in S\\ |\{u,v\}\cap \{x,z\}|=1 \end{array}}cost\left(u,v\right)\cdot cost\left(x,z\right)=\\ & & \;\sum_{\{u,v\}\in S}cost\left(u,v\right)\left[\sum_{k\in S-\{u\}}cost\left(u,k\right)+\sum_{m\in S-\{v\}}cost\left(m,v\right)-2\;cost\left(u,v\right)\right]=\\ & & \;\sum_{u\in S}TC^{2}\left(u\right)-2\sum_{\{u,v\}\in S}cost^{2}\left(u,v\right)=\sum_{u\in S}TC^{2}\left(u\right)-2\sum_{e\in E}w_{e}\cdot TC\left(e\right) \end{array}$$ (6)

The first double sum in Eq (3) can be rewritten as:

$$\begin{array}{ccc} & & \sum_{\{u,v\}\in S}\sum_{\begin{array}{c} \{x,z\}\in S\\ \{u,v\}\cap \{x,z\}=\emptyset \end{array}}cost\left(u,v\right)\cdot cost\left(x,z\right)=\\ & & \;\sum_{\{u,v\}\in S}cost\left(u,v\right)\left[TC\left(T\right)-TC\left(u\right)-TC\left(v\right)+cost\left(u,v\right)\right]=\\ & & \;TC^{2}\left(T\right)-\sum_{u\in S}TC^{2}\left(u\right)+\sum_{e\in E}w_{e}\cdot TC\left(e\right) \end{array}$$ (7)

Combining Eqs (5), (6) and (7) with Eq (3) we get the expression that appears in the definition of the theorem.

### The Common Branch Length

Let T be a phylogenetic tree and let *A*, *B* ⊆ *S* be two samples of its tips. The *Common Branch Length* (CBL) between *A* and *B* is the sum of the weights of all edges that appear both in T(A) and in T(B). Recall that T(R) denotes the smallest subtree in T that contains all the tips of a sample *R*. Therefore, the CBL between *A* and *B* is equal to:

$$\begin{array}{c} \text{CBL}\left(T,A,B\right)=\sum_{e\in T(A)\cap T(B)}w_{e}. \end{array}$$

Samples *A* and *B* may not have the same number of elements. **The CBL is analogous to the** ***Phylogenetic Diversity*** **measure for computing a diversity value between two samples of species**.

#### The Common Length Index

For a phylogenetic tree T and two samples of its tips *A*, *B* such that |*A*| = *a*, and |*B*| = *b*, we denote the standardized index of the CBL by:

$$\begin{array}{c} \text{CLI}\left(T,A,B\right)=\frac{\text{CBL}\left(T,A,B\right)-E_{\text{CBL}}\left(T,a,b\right)}{sd_{\text{CBL}}\left(T,a,b\right)}, \end{array}$$ (8)

where $E_{\text{CBL}}\left(T,a,b\right)$ and $sd_{\text{CBL}}\left(T,a,b\right)$ are respectively the expected value and the standard deviation of the CBL for all possible pairs of tip samples where one sample contains exactly *a* tips and the other contains exactly *b* tips. We call this index the *Common Length Index* of *A* and *B*. The following two theorems provide analytical expressions for the expectation and standard deviation of the CBL.

**Theorem 3**. *Let* T *be a phylogenetic tree that contains s tips, and let a, b be two positive integers such that a, b ≤ s. The expected value of the CBL among all pairs of tip samples in* T *such that one sample consists of a tips and the other sample consists of b tips, is equal to*:

ECBL(T,a,b)=∑e∈Ewe(1-(s(e)a)+(s-s(e)a)(sa))(1-(s(e)b)+(s-s(e)b)(sb))

*Proof*. The expectation of the CBL for two independtly selected samples *A* and *B* is equal to:

$$\begin{array}{c} E_{\text{CBL}}\left(T,a,b\right)=E_{\begin{array}{c} A\in \text{Sub}(S,a)\\ B\in \text{Sub}(S,b) \end{array}}\left[\sum_{e\in S}\left(w_{e}\cdot AP_{A,B}\left(e\right)\right)\right]=\sum_{e\in S}w_{e}\cdot E_{\begin{array}{c} A\in \text{Sub}(S,a)\\ B\in \text{Sub}(S,b) \end{array}}\left[AP_{A,B}\left(e\right)\right], \end{array}$$

where *AP*_*A*, *B*_(*e*) is equal to one if e∈T(A)∩T(B), otherwise is zero. The expectation of *AP*_*A*, *B*_(*e*) is equal to:

EA∈Sub(S,a)B∈Sub(S,b)[APA,B(e)]=Pr[e∈T(A)∩T(B)]=Pr[e∈T(A)]·Pr[e∈T(B)]=(1-Pr[e∉T(A)])(1-Pr[e∉T(B)])=(1-(s(e)a)+(s-s(e)a)(sa))(1--(s(e)b)+(s-s(e)b)(sb)),

and the expression for the expectation follows.

**Theorem 4**. *Let* T *be a phylogenetic tree that contains s tips, and let a, b be two positive integers such that a, b ≤ s. The standard deviation of the CBL among all pairs of tip samples in* T *such that one sample consists of a tips and the other sample consists of b tips, is equal to*:

$$\begin{array}{c} sd_{\text{CBL}}\left(T,a,b\right)=\sqrt{\sum_{e\in E}\sum_{l\in E}w_{e}\cdot w_{l}\left(1-F\left(S,e,l,a\right)\right)\left(1-F\left(S,e,l,b\right)\right)-E_{\text{CBL}}^{2}\left(T,a,b\right)}, \end{array}$$ (9)

*where*:

F(S,e,l,r)={(s(e)r)+(s-s(l)r)-(s(e)-s(l)r)(sr)ifl∈T(e).(s(l)r)+(s-s(e)r)-(s(l)-s(e)r)(sr)ife∈T(l).(s-s(e)r)+(s-s(l)r)-(s-s(e)-s(l)r)(sr)otherwise. (10)

*Proof*. The standard deviation of the CBL is equal to:

$$\begin{array}{c} \sqrt{E_{\begin{array}{c} A\in \text{Sub}(S,a)\\ B\in \text{Sub}(S,b) \end{array}}\left[{\text{CBL}}^{2}\left(T,A,B\right)\right]-E_{\text{CBL}}^{2}\left(T,a,b\right)}. \end{array}$$

In Theorem 3 we provided an expression for the expectation of the CD. It remains to derive an expression for the expectation of the squared value of this measure. We get that:

$$\begin{array}{c} E_{\begin{array}{c} A\in \text{Sub}(S,a)\\ B\in \text{Sub}(S,b) \end{array}}\left[{\text{CBL}}^{2}\left(T,A,B\right)\right]=\sum_{e\in E}\sum_{l\in E}w_{e}\cdot w_{l}\cdot E_{\begin{array}{c} A\in \text{Sub}(S,a)\\ B\in \text{Sub}(S,b) \end{array}}\left[AP_{A,B}\left(e\right)\cdot AP_{A,B}\left(l\right)\right]= \end{array}$$ (11)

$$\begin{array}{c} \sum_{e\in E}\sum_{l\in E}w_{e}\cdot w_{l}\cdot Pr\left[\left\{e,l\in T\left(A\right)\right\}\cap \left\{e,l\in T\left(B\right)\right\}\right] \end{array}$$ (12)

Events {e,l∈T(A)} and {e,l∈T(B)} are independent, so the probability value in the last sum can be rewritten as:

$$\begin{array}{c} Pr[\{e,l\in T(A)\}\cap \{e,l\in T(B)\}]=Pr[e,l\in T(A)]\cdot Pr[e,l\in T(B)]. \end{array}$$ (13)

Value Pr[e,l∈T(A)] is the probability that both edges *e*, *l* appear in T(A). Recall that T(A) is the smallest subtree of T that contains all tips in *A*. An edge *e* does not appear in T(A) if either all, or none of the elements in *A* are tips in the subtree of *e*. Given that, we get:

Pr[e,l∈T(A)]=1-Pr[(e∉T(A))∪(l∉T(A))]=1-Pr[e∉T(A)]-Pr[l∉T(A)]+Pr[(e∉T(A))∩(l∉T(A))]=1-(s(e)a)+(s-s(e)a)-(s(l)a)+(s-s(l)a)(sa)+Pr[(e∉T(A))∩(l∉T(A))] (14)

For the probability value Pr[(e∉T(A))∩(l∉T(A))] we distinguish three cases:

(I) Edge l∈T(e):
  Pr[(e∉T(A))∩(l∉T(A))]=(s-s(e)a)+(s(l)a)+(s(e)-s(l)a)(sa) (15)
(II) Edge *e* ∈ *T*(*l*). This case is symmetric to case (I):
  Pr[(e∉T(A))∩(l∉T(A))]=(s-s(l)a)+(s(e)a)+(s(l)-s(e)a)(sa) (16)
(III) Subtrees T(e) and T(l) do not contain each other:
  Pr[(e∉T(A))∩(l∉T(A))]=(s(e)a)+(s(l)a)+(s-s(e)-s(l)a)(sa) (17)

The probability value Pr[(e,l∈T(B))] can be expressed in a similar manner. The analytical expression for the standard deviation of the CBL follows by combining Eqs (11)–(17).

### Design of Algorithms

Based on the analytical expressions that we presented in the previous sections, we designed efficient algorithms for calculating the value and the standardized indices of the CD and the CBL. Next, we present in short how we designed these algorithms, and we also give a theoretical measure for their efficiency. Before continuing with the description of the algorithms, we explain standard concepts and notation from the field of Algorithms Design that we use to describe the efficiency of our algorithms.

In many applications, it is important to define a simple bound that describes the order of growth for a given function. For example, let *G* be a set of *n* real numbers. Suppose that we want to count the number of possible subsets that can be created by picking four elements of *G*. The exact number of these subsets is equal to $f\left(n\right)=\frac{1}{24}\left(n^{4}-6n^{3}+11n^{2}-6n\right)$. But, this expression is quite complicated. Suppose that we want to express how fast *f*(*n*) grows as the value of *n* increases. In that case, for large values of *n*, the term that influences the value of *f*(*n*) the most is *n*^4^. The standard way to express this in short is to say that *f*(*n*) = *O*(*n*^4^). The notation *O*(*n*^4^) is used to indicate that the value of *f*(*n*) grows roughly as fast as *n*^4^ when *n* becomes large. This is known as the *Big-Oh* notation. More formally, let *g*(*n*) and *f*(*n*) be two functions. We say that *f*(*n*) = *O*(*g*(*n*)) if there exist two positive constants *c* and *n*_0_ such that *f*(*n*)≤*c* ⋅ *g*(*n*) for every *n* ≥ *n*_0_.

In the fields of Algorithms Design, the *O*(⋅) notation is used extensively for measuring several aspects of an algorithm’s efficiency. The standard way of measuring the efficiency of an algorithm is to count the number of basic operations that would take place during its execution. When we refer to basic operations, we mean all simple mathematical operations (such as comparisons, additions, divisions), but also standard operations like initialising a variable, or assigning a value to this variable. Let A be an algorithm, and suppose we have an input dataset for this algorithm that has size *n*; for instance a phylogenetic tree T that consists of *n* elements. Instead of counting exactly the number of basic operations that take place when A processes T, we can bound this number using the *Big-Oh* notation. Ideally, we would like to prove that, for some function *g*(*n*), algorithm A takes *O*(*g*(*n*)) operations to process *any* input of size *n*. In that case, we say that the *worst case running time complexity* of A, or simply the *worst case time complexity* of this algorithm is *O*(*g*(*n*)).

Next we describe in short the algorithms that we designed for computing the standardized indices of the CD and the CBL. For each of the algorithms that we describe, we also provide a bound for its worst case time complexity. Let T be a phylogenetic tree, and let *A* and *B* be two samples of tips in T such that *A* has *a* tips and *B* has *b* tips. As described in Eqs (1) and (8), to compute the standardized index of either CD or CBL we need to calculate three values; we need to calculate the value of one of these measures for samples *A* and *B*, and we need to compute the expectation and the standard deviation of this measure among all pairs of samples in T where one sample has *a* tips and the other sample has *b* tips. Next, for each measure, we describe an algorithm for computing each of these three values.

### Algorithms for computing the CD and NRI_*β*_

#### Computing the value of the CD for a given pair of samples

To compute the value of the CD efficiently, we rewrite the expression of this measure so that it can be evaluated with a small number of basic operations. Let *e* be an edge in T, and let Num(*A*, *B*, *e*) denote the number of simple paths that connect a tip in sample *A* with a tip in sample *B*, and contain edge *e*. The value of the CD for samples *A* and *B* can be rewritten as:

$$\begin{array}{c} \text{CD}\left(T,A,B\right)=\sum_{e\in T}w_{e}\cdot \text{Num}\left(A,B,e\right). \end{array}$$ (18)

For any edge e∈T, value *Num*(*A*, *B*, *e*) is equal to:

$$\begin{array}{c} Num\left(A,B,e\right)=s_{A}\left(e\right)\cdot \left(b-s_{B}\left(e\right)\right)+s_{B}\left(e\right)\cdot \left(a-s_{A}\left(e\right)\right). \end{array}$$ (19)

Therefore, computing CD(T,A,B) boils down to computing values *s*_*A*_(*e*) and *s*_*B*_(*e*) for every edge e∈T. We can compute all these values in *O*(*n*) operations using a simple recursive algorithm; for each edge *e* that we process, we first calculate values *s*_*A*_(*e*′) and *s*_*B*_(*e*′), for every edge *e*′ ∈ *Ch*(*e*); recall that Ch(*e*) is the set of the edges that are adjacent to the child node of *e*. Then, we can calculate values *s*_*A*_(*e*) and *s*_*B*_(*e*) for *e* by simply adding the corresponding values of the edges in Ch(*e*). We can do this with only *O*(*n*) operations, by traversing the edges in T appropriately. We start from the root of T, and traverse the tree top to bottom. For each edge *e* that we encounter for the first time, we consider two cases; if *e* is adjacent to a tip, we check if this tip belongs to *A* or *B*, and we set values *s*_*A*_(*e*) and *s*_*B*_(*e*) to one or zero accordingly. Then, we move upwards in the tree and we use *s*_*A*_(*e*) and *s*_*B*_(*e*) to calculate the corresponding values for the parent edge of *e*. If *e* is not adjacent to a tip, then for every edge *l* ∈ *Ch*(*e*) we first visit the subtree of *l* and we calculate recursively the values *s*_*A*_(⋅) and *s*_*B*_(⋅) for every edge in this subtree. After calculating these values for every *l* ∈ *Ch*(*e*), we can compute *s*_*A*_(*e*) and *s*_*B*_(*e*) in *O*(*Ch*(*e*)) time by evaluating sums *s*_*A*_(*e*) = ∑_*l* ∈ *Ch*(*e*)_ *s*_*A*_(*l*) and *s*_*B*_(*e*) = ∑_*l* ∈ *Ch*(*e*)_ *s*_*B*_(*l*). Such a traversal is known as a *post-order* traversal, and requires visiting each edge in T at most two times. Since T has *O*(*n*) edges, this traversal requires *O*(*n*) operations. We also perform *O*(*Ch*(*e*)) arithmetic operations to compute *s*_*A*_(*e*) and *s*_*B*_(*e*) for every edge in T, which sums up to *O*(*n*) time operations in total. After calculating values *s*_*A*_(*e*) and *s*_*B*_(*e*), we can compute CD(T,A,B) with *O*(*n*) arithmetic operations using Eqs (18) and (19). Therefore, the total number of operations that are needed to compute the value of the CD for two tip samples in T is *O*(*n*).

#### Computing the expectation of the CD

In Theorem 1 we provide an analytical expression for the expectation of the CD. Evaluating this expression boils down to evaluating TC(T). Recall that TC(T) is equal to the sum of the costs of all simple paths that connect pairs of tips in T. We can rewrite this values as:

$$\begin{array}{c} TC\left(T\right)=\sum_{e\in T}s\left(e\right)\cdot \left(s-s\left(e\right)\right). \end{array}$$

Therefore, to compute the expectation of the CD, it remains to compute *s*(*e*) for every edge *e* in T. This can be done in *O*(*n*) operations with a post-order traversal of T, as described also for the algorithm that calculates the value of the CD for two samples. Hence, we can compute the expectation for this measure with only *O*(*n*) operations.

#### Computing the standard deviation of the CD

For the standard deviation of the CD we presented an analytical expression in Theorem 2. This expression contains sums that have *O*(*n*) terms in total. Among other terms, these sums contain values TC(T) and *TC*(*e*) for every edge *e* in T. In the algorithm that we described for computing the expectation of the CD, we showed already how we can compute TC(T) with *O*(*n*) operations. We can also compute *TC*(*e*) for every edge *e* in the tree, with *O*(*n*) operations in total. This can be done as follows. For an edge *e*, we use $w_{T}\left(e\right)$ to denote the value:

$$\begin{array}{c} w_{T}\left(e\right)=\sum_{l\in T(e)}s\left(l\right)\cdot w_{l}. \end{array}$$

For any edge *e* in the tree, value *TC*(*e*) is equal to:

$$\begin{array}{c} TC\left(e\right)=\sum_{l\in \text{Ch}(e)}TC\left(l\right)+\left(s\left(e\right)-s\left(l\right)\right)\cdot w_{T}\left(l\right)+s\left(l\right)\cdot \left(w_{T}\left(e\right)-w_{T}\left(l\right)\right). \end{array}$$ (20)

To compute the standard deviation of the CD, we perform two post-order traversals of the tree T. In the first traversal, we compute value $w_{T}\left(e\right)$ for each edge *e* based on the corresponding values of the edges in Ch(*e*). In the second traversal, we use the values $w_{T}\left(\cdot \right)$ that we just calculated to compute *TC*(*e*) for every edge in the tree. Then, using these values we can evaluate the expression in Eq (20). It takes *O*(*n*) operations to traverse the tree twice and, given values TC(T) and T(e) for every edge in T, it takes *O*(*n*) arithmetic operations to calculate the expression in Eq (20). Hence, we can compute the standard deviation of the CD with *O*(*n*) operations in total.

Using the algorithms that we describe above, we can compute the value of the CD for two samples *A* and *B*, but also the expectation and the deviation of this measure in *O*(*n*) time in the worst case. Therefore, by combining these algorithms, we can derive an algorithm that computes NRI_*β*_ in *O*(*n*) time in the worst case.

### Algorithms for computing the CBL and CLI

#### Computing the value of CBL for a given pair of samples

Recall that, for two tip samples *A* and *B*, the value of the CBL is equal to the sum of the weights of all edges *e* such that *e* belongs to both subtrees T(A) and T(B). Let *R* be a sample that consists of |*R*| = *r* tips in T. If edge *e* belongs to subtree T(R), then it holds that 0 < *s*_*R*_(*e*)<*r*. Hence, the weight of an edge *e* is counted in the value CBL(T,A,B) if 0 < *s*_*A*_(*e*)<*a* and 0 < *s*_*B*_(*e*)<*b*. Therefore, to decide for every edge e∈T whether to include *w*_*e*_ in the CBL value, we simply have to compute values *s*_*A*_(*e*) and *s*_*B*_(*e*). In the algorithm that computes the value of CD, we showed how we can compute values *s*_*A*_(*e*) and *s*_*B*_(*e*) with *O*(*n*) operations. Thus, the total number of required operations for computing the value of CBL is also *O*(*n*).

#### Computing the expectation of the CBL

Theorem 3 provides an analytical expression for the expectation of the CBL. This expression consists of a sum with *O*(*n*) terms, and can be evaluated with *O*(*n*) arithmetic operations, given that we have already computed value *s*(*e*) for each edge *e* in the tree. When describing an algorithm that calculates the expectation of the CD, we showed how we can compute these values with *O*(*n*) operations. Therefore, we can compute the expectation of the CBL in *O*(*n*) time.

#### Computing the standard deviation of the CBL

The standard deviation of the CBL can be calculated using the expression that we provide in Theorem 4. This expression contains a sum of *O*(*n*^2^) terms. Evaluating this sum directly takes *O*(*n*^2^) operations, which can be inefficient in practice.

In a previous paper, we presented a technique for computing similar expressions [13]. We can use this technique for evaluating the sum in Eq (9). As we presented in our previous work, this technique is quite efficient when T is relatively balanced. More precisely, the worst case time complexity of this algorithm is O(SI(T)), where SI(T) denotes the Sackin’s Index of a tree T. The Sackin’s Index of T is equal to the sum of the depths of all tips in T. The depth of a tip *v* is equal to the number of edges that appear on the simple path between *v* and the root of the tree. For a perfectly balanced tree that consists of *n* nodes, the depth of each tip is *O*(log(*n*)). In this case, the value of of the Sackin’s Index is equal to *O*(*n* log *n*). However, if the tree is skewed, there may exist many tips with depth close to *n* and therefore the value of the Sackin’s Index is *O*(*n*^2^). Hence, in the worst case, the algorithm that we consider for computing the standard deviation of the CBL takes quadratic time with respect to the size of the input tree. In practice, phylogenetic trees are relatively balanced, and therefore the proposed algorithm is quite efficient. In the next section, we provide evidence for this argument; there we present experiments that we conducted using our algorithms on large tress.

## Results and Applications

### Experimental Evaluation

We implemented all the algorithms that we present in this paper, and we measured their performance in practice. Our implementations are developed in C++, and are publicly available through the software package PhyloMeasures [24]. This package provides functions for computing the value and the standardized indices of several phylogenetic biodiversity measures, and it is available both as an R package and a C++ library.

For the experiments that we conducted, we used a large phylogenetic tree from which we extracted several subtrees of several sizes. The tree that we used was constructed by Goloboff et. al [25]. This is the largest evolutionary tree of eukaryotic organisms that has been so far constructed from molecular and morphological data. It consists of 71,181 tips and 83,751 nodes in total. This tree is unrooted; for the needs of our experiments we picked arbitrarily an internal node and used this as the root. We call this dataset the eukaryotes dataset.

**Fig 1:**
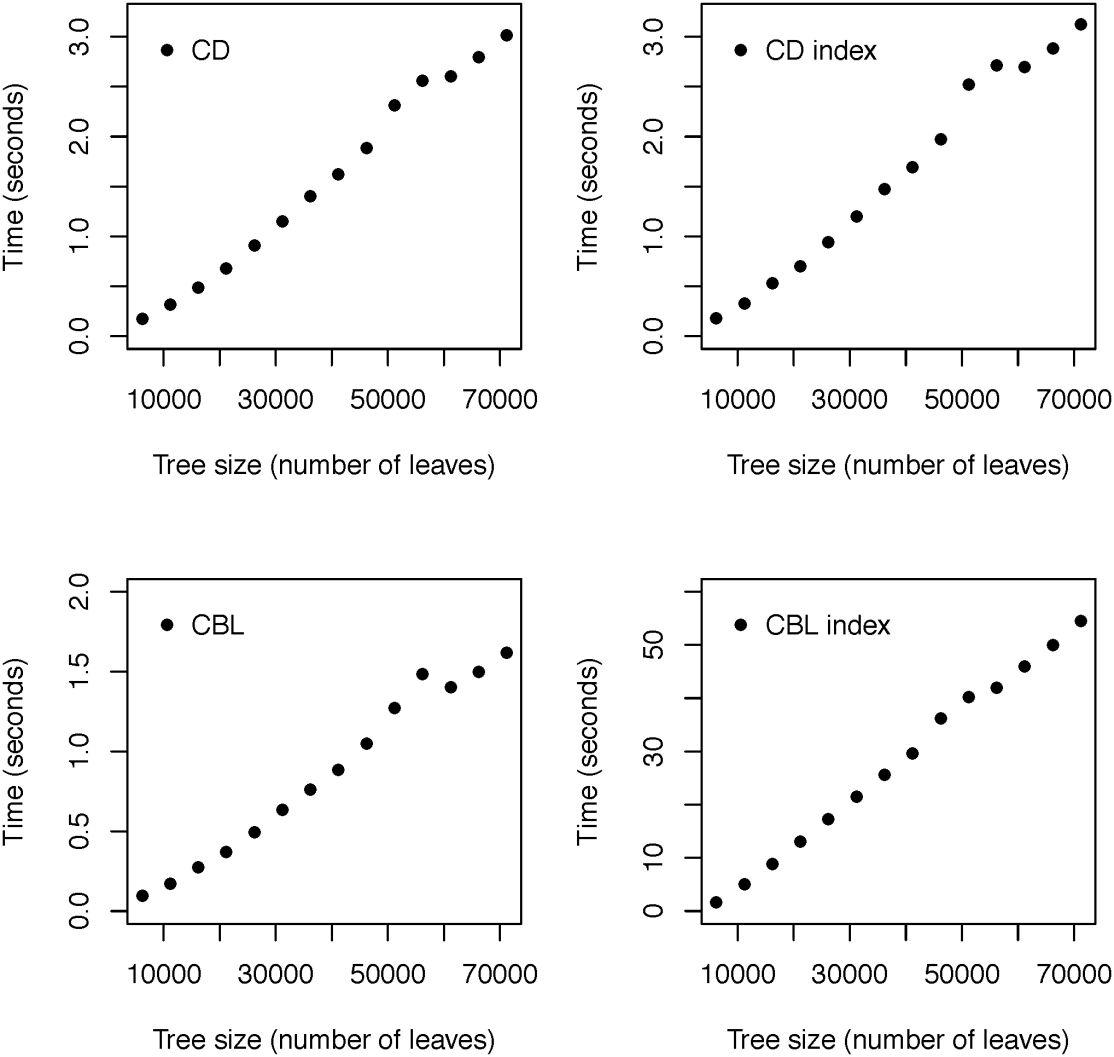
Running times of implemented algorithms computing values and standardized indices for CD and CBL. For each implementation and for each tree size, the figures illustrate the time that it takes for the function to process a set of one hundred samples.

From the eukaryotes we extracted fourteen trees, each tree having 5,000*k* + 1181 tips with *k* ∈ {1, 2, …, 14}. These subtrees were produced by successively pruning chunks of 5,000 leaves from the eukaryotes tree. We represent the set of these trees by EK. Therefore, for any two trees $T,T^{\prime }\in$ EK we have that either T is a subtree of $T^{\prime }$, or vice versa. For each tree T∈ EK we produced one hundred samples of tips with sizes s(T)/k with *k* ranging from one to a hundred. We denote this set of samples by samples (T). For every T∈ EK we executed the algorithms we implented for computing the values and the standardized indices of the CD and the CBL. For each algorithm and for each tree T∈ EK, we measured the execution time for processing all samples in samples (T). The results of these experiments are illustrated in Fig 1. The experiments were performed using the R version of the PhyloMeasures package on a computer with an Intel core i5-2430M processor. This is a four-core CPU with 2.40GHz per core. The main memory of this computer is 7.8 Gigabytes. Our implementations run on a Linux Ubuntu operating system, release 12.04. The experiments were executed using R version 3.1.2 (Pumpkin Helmet).

All of the examined implementations run very fast even for the largest trees in EK. For the complete eukaryotes tree that consists of 71,181 tips, the algorithm that computes the value of the CD takes 3.01 seconds to process one hundred samples, the algorithm that computes the CBL value takes 1.61 seconds, and the algorithms that compute the standardized indices of the CD and the CBL take 3.12 and 54.52 seconds respectively. Note that these are the running times that each program takes for computing the results for one hundred samples of species. The program that computes the index of the CBL runs slower than the other programs, yet we see that it is still quite efficient. It seems that, for the datasets that we used, its execution time does not scale as a quadratic function of the tree size.

### Applications

To illustrate an application of the algorithms, we used their PhyloMeasures implementation to calculate CBL and standardized CBL for mammals globally. We used range maps from the IUCN and rasterized them on a Behrmann equal area grid with a resolution of 193km (at 30N or S), corresponding roughly to two degrees. Each of the resulting grids consists of 71 × 180 cells, which means 12780 cells in total for each grid.

**Fig 2:**
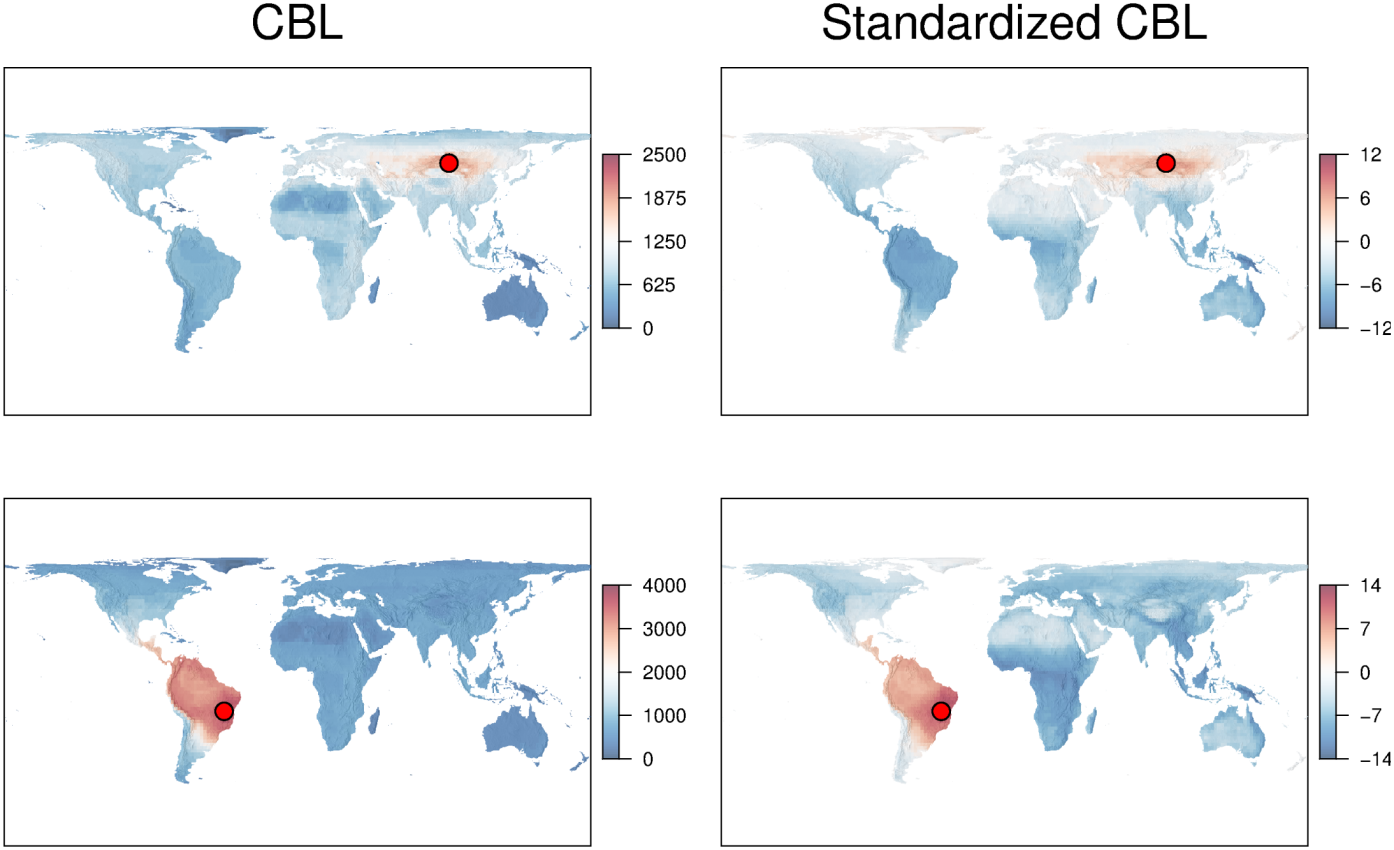
Maps of phylogenetic similarity of mammal assemblages of focal cells (red dot), compared to all other locations in the world. Similarity was calculated as Common Branch Length (CBL), or its richness-standardized version, for two different focal cells.

We combined these grids with the phylogeny of Bininda-Emonds et al [26]; this is a tree that portrays the phylogenetic relations between all mammal species. The number of leaf nodes in this tree is 4,510. Using this tree, for each grid that we created we then calculated CBL and standardized CBL between a focal cell and all other cells in the world. The results are spatial maps of the phylogenetic similarity between the focal location and all other sites. The constructed spatial maps are illustrated in Fig 2. In Central Asia, for example, there is evidence for a broad longitudinal band of high similarity, with more rapid turnover along the latitudinal gradient, while phylogenetic similarity is fairly high throughout northern South America, but declines rapidly towards the south of the continent.

Such maps can provide a detailed picture of how phylogenetic similarity changes among species assemblages over geographic space. However, constructing high resolution maps of this kind is practically infeasible without efficient algorithms that compute beta-diversity values, and especially the standardised version of these values. Using our implementations, we computed the exact standardized and non-stanndardized CBL values for these maps in one and a half minutes in total. On the other hand, it would take several days to execute these computations with previously existing software, which would also provide these values only approximately.

## Conclusion

Phylogenetic beta diversity metrics are widely and increasingly used in ecology, but are often quite slow to compute. In addition, the relationship between species richness of the samples and the beta diversity metrics is often ignored. These problems are related, as computing the dependence of a metric on species richness is even more computationally intensive using traditional approaches, than is computing a single beta diversity value. Here, we propose solutions to these problems, providing 1) efficient algorithms for computing phylogenetic beta diversity metrics, and 2) algorithms to efficiently and exactly calculate their moments, allowing a simple standardization for species richness. We expect that these algorithms will significantly ease the computational burdens of researchers and lead to wider adoption of phylogenetic beta diversity metrics.

## References

[1] Whittaker RH. Vegetation of the Siskiyou mountains, Oregon and California. Ecological Monographs. 1960; 30: 279–338. 10.2307/1948435

[2] Cody ML. Towards a theory of continental species diversities: bird distributions over mediterranean habitat gradients In: Cody ML, Diamond JM, editors. Ecology and evolution of communities. Harvard University Press; 1975 pp. 214–257.

[3] Meynard CN, Devictor V, Mouillot D, Thuiller W, Jiguet F, Mouquet N. Beyond taxonomic diversity patterns: how do *α*, *β* and *γ* components of bird functional and phylogenetic diversity respond to environmental gradients across France? Global Ecology and Biogeography. 2011; 20:893–903. 10.1111/j.1466-8238.2010.00647.x

[4] Swenson NG, Erickson DL, Mi X, Bourg NA, Forero-Montaña J, Ge X, Howe R, Lake JK, Liu X, Ma K, Pei N, Thompson J, Uriarte M, Wolf A, Wright SJ, Ye W, Zhang J, Zimmerman JK, Kress WJ. Phylogenetic and functional alpha and beta diversity in temperate and tropical tree communities. Ecology. 2012; 93:S112–S125. 10.1890/11-1180.1

[5] Graham CH, Fine PVA. Phylogenetic beta diversity: linking ecological and evolutionary processes across space and time. Ecology Letters. 2008; 11: 1265–1277. 10.1111/j.1461-0248.2008.01256.x 19046358

[6] Holt BG, Lessard JP, Borregaard MK, Fritz SA, Araújo MB, Dimitrov D, Fabre PH, Graham CH, Graves GR, Jønsson KA, Nogués-Bravo D, Wang Z, Whittaker RJ, Fjeldså J, Rahbek C. An update of Wallace’s zoogeographic regions of the world. Science. 2013;339, no. 6115: 74–78. 10.1126/science.1228282 23258408

[7] Qian H, Swenson NG, Zhang J. Phylogenetic beta diversity of angiosperms in North America. Global Ecology and Biogeography. 2013; 22:1152–1161. 10.1111/geb.12076

[8] Peixoto FP, Braga PHP, Cianciaruso MV, Diniz-Filho JAF, Brito D. Global patterns of phylogenetic beta diversity components in bats. Journal of Biogeography. 2014; 41:762–772. 10.1111/jbi.12241

[9] Swenson NG. Phylogenetic beta diversity metrics, trait evolution and inferring the functional beta diversity of communities. PLoS ONE. 2011; 6: e21264 10.1371/journal.pone.0021264 21731685 PMC3123305

[10] Steel M. Tools to construct and study big trees: a mathematical perspective In: Hodkinson T, Parnell J, Waldren S, editors. Reconstructing the tree of life: taxonomy and systematics of species rich taxa. CRC Press; 2007 pp. 97–112.

[11] O’Dwyer JP, Kembel SW, Green JL. Phylogenetic diversity theory sheds light on the structure of microbial communities. PLoS Computational Biology. 2012; 8: e1002832 10.1371/journal.pcbi.1002832 23284280 PMC3527210

[12] Tsirogiannis C, Sandel B, Cheliotis D. Efficient computation of popular phylogenetic tree measures. Lecture Notes on Computer Science; 2012; 7534: 30–43. 10.1007/978-3-642-33122-0_3

[13] Tsirogiannis C, Sandel B, Kalvisa A. New algorithms for computing phylogenetic biodiversity. Algorithms in Bioinformatics. 2014; LNCS 8701:187–203.

[14] Nipperess DA, Matsen FA IV. The mean and variance of phylogenetic diversity under rarefaction. Methods in Ecology and Evolution. 2013; 4: 566–572. 10.1111/2041-210X.12042 23833701 PMC3699894

[15] Chao A, Chiu CH, Hsieh TC, Davis T, Nipperess DA, Faith DP. Rarefaction and extrapolation of phylogenetic diversity. Methods in Ecology and Evolution. 2015; 6: 380–388. 10.1111/2041-210X.12247

[16] Webb CO, Ackerly DD, McPeek MA, Donoghue MJ. Phylogenies and community ecology. Annual Review of Ecology and Systematics. 2002;33: 475–505. 10.1146/annurev.ecolsys.33.010802.150448

[17] Kembel SW, Cowan PD, Helmus MR, Cornwell WK, Morlon H, Ackerly DD, Blomberg SP, Webb CO. Picante: R tools for integrating phylogenies and ecology. Bioinformatics. 2010; 26:1463–1464. 10.1093/bioinformatics/btq166 20395285

[18] Graham CH, Parra JL, Rahbek C, McGuire JA. Phylogenetic structure in tropical hummingbird communities. Proceedings of the National Academy of Science. 2009; 106:19673–19678. 10.1073/pnas.0901649106 PMC2780942 19805042

[19] Leprieur F, Albouy C, De Bortoli J, Cowman PF, Bellwood DR, Mouillot D. Correction: Quantifying phylogenetic beta diversity: distinguishing between ‘true’ turnover of lineages and phylogenetic diversity gradients. PLoS ONE. 2012; 7(10):. 10.1371/journal.pone.0042760 22912736 PMC3422232

[20] Pellissier L, Ndiribe C, Dubuis A, Pradervand JN, Salamin N, Guisan A, Rasmann S. Turnover of plant lineages shapes herbivore phylogenetic beta diversity along ecological gradients. Ecology letters 2013; 16(5), 600–608. 10.1111/ele.12083 23448096

[21] Feng G, Mi X, Eiserhardt WL, Jin G, Sang W, Lu Z, Wang X, Li X, Li B, Sun I, Ma K, Svenning J-C. Assembly of forest communites across East Asia—insights from phylogenetic community structure and species pool scaling. Scientific Reports. 2015; 5: 9337 10.1038/srep09337 25797420 PMC4369734

[22] Ricotta C, Bacaro G, Pavoine S. A cautionary note on some phylogenetic dissimilarity measures. Journal of Plant Ecology. 2015; 8(1):12–16. 10.1093/jpe/rtu008

[23] Eiserhardt WL, Svenning J-C, Borchsenius F, Kristiansen T, Balslev H. Separating environmental and geographical determinants of phylogenetic community structure in Amazonian palms (Arecaceae). Botanical Journal of the Linnean Society. 2013; 171: 244–259. 10.1111/j.1095-8339.2012.01276.x

[24] Tsirogiannis C, Sandel B. PhyloMeasures: a package for computing phylogenetic biodiversity measures and their statistical moments. Ecography. 2015;. 10.1111/ecog.01814

[25] Goloboff PA, Catalano SA, Mirandeb JM, Szumika CA, Ariasa JS, Kallersjoc M, Farris JS. Phylogenetic analysis of 73060 taxa corroborates major eukaryotic groups. Cladistics. 2009; 25:211–230. 10.1111/j.1096-0031.2009.00255.x 34879616

[26] Bininda-Emonds ORP, Cardillo M, Jones KE, MacPhee RDE, Beck RMD, Grenyer R, Price SA, Vos RA, Gittleman JL, Purvis A. The delayed rise of present-day mammals. Nature. 2007; 446:507–512. 10.1038/nature05634 17392779

